# Sex-Specific Associations of *MIR137* Polymorphisms With Schizophrenia in a Han Chinese Cohort

**DOI:** 10.3389/fgene.2021.627874

**Published:** 2021-02-23

**Authors:** Jingwen Yin, Xudong Luo, Qian Peng, Susu Xiong, Dong Lv, Zhun Dai, Jiawu Fu, Ying Wang, Yaxue Wei, Chunmei Liang, Xusan Xu, Dandan Zhang, Lulu Wang, Dongjian Zhu, Xia Wen, Xiaoqing Ye, Zhixiong Lin, Juda Lin, You Li, Jiafeng Wang, Guoda Ma, Keshen Li, Yajun Wang

**Affiliations:** ^1^Department of Psychiatry, Affiliated Hospital of Guangdong Medical University, Zhanjiang, China; ^2^Center for Cognitive and Brain Sciences, Institute of Collaborative Innovation, University of Macau, Taipa, China; ^3^Department of Psychology, Faculty of Social Sciences, University of Macau, Taipa, China; ^4^Maternal and Children’s Health Research Institute, Shunde Maternal and Children’s Hospital, Guangdong Medical University, Foshan, China; ^5^Institute of Neurology, Guangdong Medical University, Zhanjiang, China; ^6^Stem Cell Research and Cellular Therapy Center, Affiliated Hospital of Guangdong Medical University, Zhanjiang, China; ^7^Guangdong Key Laboratory of Age-Related Cardiac and Cerebral Diseases, Affiliated Hospital of Guangdong Medical University, Zhanjiang, China

**Keywords:** *MIR137*, polymorphism, rs2660304, rs1198588, schizophrenia

## Abstract

**Objective**: To investigate the effects of microRNA-137 (*MIR137*) polymorphisms (rs1198588 and rs2660304) on the risk of schizophrenia in a Han Chinese population.

**Methods**: Schizophrenia was diagnosed according to the DSM-5. Clinical symptoms and cognitive functions were assessed with the Positive and Negative Symptom Scale (PANSS) and Brief Assessment of Cognition in Schizophrenia (BACS), respectively. The polymorphisms were genotyped by improved multiplex ligation detection reaction (iMLDR) technology in 1,116 patients with schizophrenia and 1,039 healthy controls.

**Results**: Significant associations were found between schizophrenia and *MIR137* in the distributions of genotypes (*p* = 0.037 for rs1198588; *p* = 0.037 for rs2660304, FDR corrected) and alleles (*p* = 0.043 for rs1198588; *p* = 0.043 for rs2660304, FDR corrected) of two SNPs. When the population was stratified by sex, we found female-specific associations between *MIR137* and schizophrenia in terms of genotype and allele distributions of rs1198588 (*χ*^2^ = 4.41, *p* = 0.036 and *χ*^2^ = 4.86, *p* = 0.029, respectively, FDR corrected) and rs2660304 (*χ*^2^ = 4.74, *p*=0.036 and *χ*^2^ = 4.80, *p* = 0.029, respectively, FDR corrected). Analysis of the *MIR137* haplotype rs1198588-rs2660304 showed a significant association with schizophrenia in haplotype T-T [*χ*^2^ = 4.60, *p* = 0.032, OR = 1.32, 95% CI (1.02–1.70)]. Then, significant female-specific associations were found with the haplotypes T-T and G-A [*χ*^2^ = 4.92, *p* = 0.027, OR = 1.62, 95% CI (1.05–2.50); *χ*^2^ = 4.42, *p* = 0.035, OR = 0.62, 95% CI (0.39–0.97), respectively]. When the TT genotype of rs1198588 was compared to the GT+GG genotype, a clinical characteristics analysis also showed a female-specific association in category instances (*t* = 2.76, *p* = 0.042, FDR corrected).

**Conclusion**: The polymorphisms within the *MIR137* gene are associated with susceptibility to schizophrenia, and a female-specific association of *MIR137* with schizophrenia was reported in a Han Chinese population.

## Introduction

Based on converging evidence from several research disciplines, it has been generally accepted that schizophrenia is a disease characterized by a nervous system dysfunction caused by the “accumulation” of dysregulation events in multiple candidate genes (Zhu et al., 2016). These susceptible genes can act on several levels, from the gene product itself through molecular, cellular, and tissue levels, all the way to complex phenotypes such as psychotic symptoms and cognitive deficits (Dobbyn et al., 2018). Systematically understanding how genes act to confer risk for schizophrenia is an obvious strategy in biological psychiatry.

miR-137 is highly expressed in the brain and has important roles in neurogenesis, neuronal maturation, and signal transduction (Guella et al., 2012; Wang et al., 2020). The dysregulation of miR-137 usually causes micro-abnormalities in neural structure and function, which might lead to brain function disorders, including schizophrenia. Clinical research further provided evidence of 2.63-fold higher miR-137 expression in the peripheral blood of schizophrenia patients compared with healthy controls (Wu et al., 2016). Transgenic mouse overexpression of *MIR137* in the whole brain induces several phenotypes that are relevant to aspects of psychiatric disorders, including sensory gating deficits and social and cognitive deficits (Arakawa et al., 2019). In addition, *MIR137* has also been identified as specifically regulating genes with replicated evidence for a role in schizophrenia, most notably N-methyl-D-aspartate (NMDA) receptor (Strazisar et al., 2014), glutamate ionotropic receptor NMDA type subunit 2A (Grin2A; Zhao et al., 2013), GluA1 subunit of alpha-amino-3-hydroxy-5-methyl-4-isoxazole propionic acid receptors (AMPARs; Olde Loohuis et al., 2015), zinc finger binding protein 804A (ZNF804A; Kim et al., 2012), transcription factor 4 (TCF4; Wright et al., 2013), brain-derived neurotrophic factor (BDNF; Hill et al., 2014; Thomas et al., 2017), and glyoxalase 1 (Hambsch, 2011; Bangel et al., 2015; Lv et al., 2018). Overall, *MIR137* appears to be an integral part of the biological pathways of schizophrenia and a component of the genetic architecture of the complex phenotypes of schizophrenia.

Genome-wide association studies (GWAS) have suggested that rs1625579 of *MIR137* was the strongest locus associated with schizophrenia (Ripke et al., 2011). In our previous study, rs1625579 was reported as a risk SNP in the southern Chinese population (Ma et al., 2014). SNP rs2660304, located in the promoter (4.0 kb upstream) of the primary transcript sequence of the *MIR137* host gene, has strong linkage disequilibrium (LD) with rs1625579. Siegert (2015) and Warburton et al. (2016) observed increased transcriptional and expression levels of miR-137 in the minor allele-carrying cells of rs2660304 compared to the major allele. Rs1198588 is also a GWAS-suggested locus that lies within a noncoding genomic but open chromatin region and can alter miR-137 expression in an epigenetic manner (Ripke et al., 2013; Siegert et al., 2015; Forrest et al., 2017). To further assess the associations of *MIR137* SNPs with the risk of schizophrenia, we conducted a case-control study in the Han Chinese population.

## Subjects and Methods

### Subjects

A total of 1,116 schizophrenic patients and 1,039 healthy controls were recruited from the Affiliated Hospital of Guangdong Medical University, and all patients were from the Han population. All patients underwent a series of standardized examinations, including family history, an evaluation regarding a wide range of drugs and alcohol, a physical and neurological examination, and laboratory testing, to eliminate those with a drug-induced psychotic disorder or psychosis resulting from general medical conditions. The diagnosis was made independently by two well-trained senior psychiatrists based on the Diagnostic and Statistical Manual of Mental Disorders, Fifth Edition (DSM-5). Psychiatric symptoms were assessed using a structured interview by the Positive and Negative Symptom Scale (PANSS). Evaluations of the cognitive function of patients were performed by the Brief Assessment of Cognition in Schizophrenia (BACS; Keefe et al., 2004). The BACS includes the following six parts: working memory measured by the digit sequencing task, verbal memory measured by list learning, motor speed measured by the token motor task, reasoning and problem solving measured by the Tower of London, attention and processing speed, and verbal fluency measured by symbol coding. None of the healthy controls had a personal or family history of major mental disease, severe physical illness, or substance abuse. The study protocol was approved by the ethics committee of the Affiliated Hospital of Guangdong Medical University, and written informed consent was obtained from subjects or immediate family members.

### DNA Extraction and Genotyping

Genomic DNA from EDTA-anticoagulated peripheral blood was extracted using the TIANamp Blood DNA Kit (Tiangen Biotech, Beijing, China). The rs1198588 and rs2660304 SNPs were genotyped using the improved multiplex ligation detection reaction (iMLDR) method (Genesky Biotechnologies Inc., Shanghai, China) as described previously (Xu et al., 2018; Yin et al., 2019). The primer information for the multiplex polymerase chain reaction and ligation is described in Supplementary Table S1.

### Statistical Analysis

Statistical analysis was performed by SPSS 21.0 software. Descriptive data are presented as the mean ± standard deviation (SD) and frequencies (%) as appropriate. Pearson’s chi-square test was used to assess Hardy-Weinberg equilibrium (HWE) and the differences in genotypic and allelic distributions between patients and controls. Then, generalized odds ratios (ORs) with 95% confidence intervals (CIs) of the alleles were calculated. In the association analysis between genotypes and clinical characteristics, categorical variables were compared by Pearson’s chi-square tests, and continuous variables were compared by Student’s t test between two independent groups. The normalized linkage disequilibrium coefficient (D') and squared correlation coefficient (*r*^2^) were used to measure LD. The LD status and haplotype analysis were determined using Haploview 4.2 software. Only those haplotypes with frequencies greater than 3% were further analyzed. Multiple comparisons were performed with false discovery rate (FDR) correction in R and R studio software. Power calculations were performed using QUANTO 1.2 software. A value of 0.05 was used as a threshold for statistical significance after correction for multiple comparisons.

## Results

There were no statistically significant differences in the age and sex distribution of the *MIR137* polymorphisms between the patient group and the control group, as shown in Supplementary Table S2. The distributions of the two loci in the controls were consistent with Hardy-Weinberg equilibrium, as shown in Table 1. Significant differences were observed in the genotype and allele distributions of rs1198588 (*χ*^2^ = 4.37, *p* = 0.037 and *χ*^2^ = 4.57, *p* = 0.043, respectively, FDR corrected) and rs2660304 (*χ*^2^ = 4.42, *p* = 0.037 and *χ*^2^ = 4.10, *p* = 0.043, respectively, FDR corrected) of *MIR137*, as shown in Table 1.

**Table 1:** Genotype and allele frequencies of SNPs in *MIR137* in schizophrenic patients and controls.

Groups	*N*	Genotype N (%)			*χ*^2*^	*p*	*P*_FDR_	Allele N (%)		*χ*^2^	*p*	*P*_FDR_	OR (95% CI)	*P*_HWE_
rs1198588		TT	TA	AA				T	A					
**Total**														
Patients	1,116	1,005 (90.05)	106 (9.50)	5 (0.45)	4.37	0.036	0.037	2,116 (94.80)	116 (5.19)	4.57	0.033	0.043	1.32 (1.02–1.69)	
Controls	1,039	906 (87.20)	126 (12.13)	7 (0.67)				1938 (93.26)	140 (6.74)					0.26
**Male**														
Patients	709	632 (89.14)	73 (10.30)	4 (0.56)	1.15	0.283	0.288	1,337 (94.29)	81 (5.71)	1.12	0.289	0.334	1.19 (0.87–1.63)	
Controls	619	540 (87.24)	75 (12.12)	4 (0.65)				1,155 (93.30)	83 (6.70)					0.43
**Female**														
Patients	407	373 (91.65)	33 (8.10)	1 (0.25)	4.41	0.036	0.036	779 (95.70)	35 (4.30)	4.86	0.027	0.029	1.62 (1.05–2.50)	
Controls	420	366 (87.14)	51 (12.14)	3 (0.71)				783 (93.21)	57 (6.79)					0.41
**rs2660304**		**TT**	**GT**	**GG**				**T**	**G**					
**Total**														
Patients	1,116	1,010 (90.50)	101 (9.05)	5 (0.45)	4.42	0.035	0.037	2,121 (95.03)	111 (4.97)	4.10	0.043	0.043	1.31 (1.01–1.69)	
Controls	1,039	911 (87.68)	123 (11.84)	5 (0.48)				1945 (93.60)	133 (6.40)					0.70
**Male**														
Patients	709	634 (89.42)	71 (10.01)	4 (0.56)	1.13	0.288	0.288	1,339 (94.43)	79 (5.57)	0.93	0.334	0.334	1.17 (0.85–1.61)	
Controls	619	542 (87.56)	74 (11.96)	3 (0.48)				1,158 (93.54)	80 (6.46)					0.78
**Female**														
Patients	407	376 (92.38)	30 (7.37)	1 (0.25)	4.74	0.029	0.036	782 (96.07)	32 (3.93)	4.80	0.029	0.029	1.65 (1.05–2.58)	
Controls	420	369 (87.86)	49 (11.66)	2 (0.48)				787 (93.69)	53 (6.31)					0.79

*P*_FDR_: *p* values have been corrected by FDR correction.

Furthermore, to examine the possible potential effect of sex on the associations between the SNPs and schizophrenia, we stratified the participants into male and female subgroups. Significant differences were maintained in the genotype and allele distributions of rs1198588 (*χ*^2^ = 4.41, *p* = 0.036 and *χ*^2^ = 4.86, *p* = 0.029, respectively, FDR corrected) and rs2660304 (*χ*^2^ = 4.74, *p*=0.036 and *χ*^2^ = 4.80, *p* = 0.029, respectively, FDR corrected) in the female subgroup but vanished in the male subgroup (*p* > 0.05; Table 1).

LD analysis was performed in two loci of *MIR137*. Strong LD was observed in rs1198588 and rs2660304 (D' = 0.996, *r*^2^ = 0.94). The association of risk SNPs with schizophrenia was further supported by the results of the haplotype analysis, and the corresponding results are shown in Table 2. The frequency of the T-T haplotype (rs2660304-rs1198588) was significantly higher in the schizophrenia patients than in the controls [*χ*^2^ = 4.60, *p* = 0.032, OR = 1.32, 95% CI (1.02–1.70)]. When the population was stratified by sex, a significant association was also evidenced only in the female subgroup, wherein the overall association was significant [*χ*^2^ = 4.92, *p* = 0.027, OR = 1.62, 95% CI (1.05–2.50)], and no such association was observed in the male subgroup [*χ*^2^ = 1.12, *p* = 0.289, OR = 1.19, 95% CI (0.87–1.63)]. Thus, the haplotype was shown to be a female-specific risk factor for schizophrenia. Furthermore, our results showed that the G-A haplotype was a protective factor against schizophrenia only in the female group and not in the male group [*χ*^2^ = 4.42, *p* = 0.035, OR = 0.62, 95% CI (0.39–0.97); Table 2].

**Table 2:** Haplotype analysis of SNPs in *MIR137* in schizophrenic patients and controls.

rs2660304-rs1198588		Frequency	Schizophrenia		Controls		*χ*^2^	*p*	OR (95% CI)
			N	%	N	%			
**Total**									
	T–T	0.94	2,116	94.80%	1936	93.26%	4.60	0.032	1.32 (1.02–1.70)
	G-A	0.06	111	4.97%	132	6.36%	3.88	0.049	0.77 (0.59–0.99)
**Male**									
	T–T	0.94	1,337	94.29%	1,155	93.30%	1.12	0.289	1.19 (0.87–1.63)
	G-A	0.06	79	5.57%	80	6.46%	0.93	0.334	0.85 (0.62–1.18)
**Female**									
	T-T	0.94	779	95.70%	781	93.20%	4.92	0.027	1.62 (1.05–2.50)
	G-A	0.05	32	3.93%	52	6.21%	4.42	0.035	0.62 (0.39–0.97)

N, number; OR, odds ratio; 95% CI, 95% confidence interval. The *χ*^2^ test was performed by one haplotype compared with others.

Using the BACS, we analyzed the neurocognitive function of the patients, and significant differences were found in category instances when the TT genotypes of rs1198588 and rs2660304 were compared to the GT+GG genotype in the female group, but only the comparison in rs1198588 survived after FDR correction (*t* = 2.76, *p* = 0.042). Otherwise, differences were shown in token motor task scores in males when the TT genotype was compared to G carriers in both rs1198588 and rs2660304, but these differences did not survive multiple comparison correction (shown in Supplementary Table S3). Our study further analyzed the distribution of other clinical characteristics among different genotypes in the patients, but there were no significant differences in age of onset, family history, or PANSS scores (*p* > 0.05).

## Discussion

The *MIR137* host gene was most strongly associated with schizophrenia in GWAS (Ripke et al., 2011). *MIR137* is highly and specifically expressed in the brain (Supplementary Figure S1; data were downloaded from the GTEx database) and regulates neural structure and function (Yin et al., 2014; Mahmoudi and Cairns, 2017). Increasing evidence has shown that *MIR137* and its gene regulatory network may be involved in the genetic and biological basis of schizophrenia and have been associated with phenotypes in schizophrenia, such as age of onset (Lett et al., 2013) and brain structure and function (Whalley et al., 2012; Lett et al., 2013; Vogel et al., 2018; Zhang et al., 2018). Rs1198588 and rs2660304 are located at 41.1 and 4.0 kb, respectively, upstream of the *MIR137* host gene region and are related to the transcript regulation and expression of miR-137 (Warburton et al., 2016; Forrest et al., 2017). Rs2660304 resulted in downregulation of reporter gene expression in a tissue culture model (Warburton et al., 2016; Tian et al., 2019). The LD analysis indicated that rs1198588 and rs2660304 might be proxies of rs1625579. In our sample, rs1625579 also showed LD with rs1198588 (*D'* = 0.99, *r*^2^ = 0.94) and rs2660304 (*D'* = 1, *r*^2^ = 0.99; calculated by combining the data set with previous results). We performed a case-control study to primarily investigate rs1198588 and rs2660304 in the risk of schizophrenia in a Han population from southern China. The results indicated that both are associated with the risk of schizophrenia. At present, the biological mechanisms of *MIR137* rs1198588 and rs2660304 polymorphisms are still unclear, but the results of our case-control study provide convincing evidence for the association between *MIR137* polymorphisms and schizophrenia. Power analysis showed that the sample has 95% power to detect relative risk with an assumed odds ratio of 1.5 at the 0.05 level.

Interesting results in our study were sex differences in the *MIR137* genotype and haplotype, which were relatively pronounced in females but not significant in males. It is perhaps not surprising, considering the substantial evidence for sex differences in the etiology and pathogenesis of schizophrenia (Aleman et al., 2003; Kulkarni et al., 2012). Previous studies have presented female-specific effects on *MIR137* and target genes of *MIR137*. Kandratsenka et al. (2018) reported female-specific effects on negative symptom scores and the total PANSS scores of the rs1625579 polymorphism of *MIR137* in a Belarusian population. Considering the high LD in rs1625579 and rs1198588, the sex-specific differences with rs1198588 failed to be repeated in the PANSS scores in our sample, but differences were found in cognitive function. A possible explanation might be the heterogeneity in symptoms and genetic distribution related to ethnicity. Interestingly, Zhang et al. (2011) provided evidence that ZNF804A, a target gene of *MIR137*, also showed a female-specific association with schizophrenia. In addition, Glo-1, another target gene of *MIR137*, has also been reported to be involved in the regulation of sex hormones. This evidence suggests a possible pathway by which sex hormones modulate the expression of miR-137 and its targets, which may account for the observed sex-specific effects. Functional and expression studies are necessary to further clarify this hypothesis, and the result needs to be replicated in different independent samples, since there is a possibility of population bias. The limited sample size for the individual sexes was not large enough, and the sex-genotype interaction has not been formally tested.

The present study has several potential limitations. First, the sample in this study was limited to the Han Chinese population. Further investigations of the association between *MIR137* polymorphisms and schizophrenia need to be carried out in different ethnic populations. Second, we observed that differences between genotypes and haplotype frequency distributions were common, specifically among females in our study groups. Thus, a further functional analysis is needed to clarify how variants in *MIR137* play a female-specific role in the pathogenesis of schizophrenia.

In conclusion, to our knowledge, this study is the first to show that *MIR137* SNPs are significantly associated with schizophrenia in the Han Chinese population. Moreover, our study provides further evidence for a female-specific influence of the gene on the development of schizophrenia.

## Data Availability Statement

The datasets presented in this study can be found in online repositories. The names of the repository/repositories and accession number(s) can be found in the article/Supplementary Material.

## Ethics Statement

The studies involving human participants were reviewed and approved by Ethics Committee of Guangdong Medical University. The patients/participants provided their written informed consent to participate in this study.

## Author Contributions

All authors listed have made a substantial, direct and intellectual contribution to the work, and approved it for publication.

### Conflict of Interest

The authors declare that the research was conducted in the absence of any commercial or financial relationships that could be construed as a potential conflict of interest.
