# Supplementary material for: Sex-Specific Associations of MIR137 Polymorphisms With Schizophrenia in a Han Chinese Cohort
Source: Front Genet. 2021 Feb 23;12:627874. doi: 10.3389/fgene.2021.627874 (PMC7942225; doi:10.3389/fgene.2021.627874)
Supplement: Supplementary file 1 [file Table_1.docx]

Supplementary Material

## Table S1. SNP information and oligonucleotide sequences used for *MIR137* genotyping.

| SNP | Purpose | | Sequence (5’→3’) |
| --- | --- | --- | --- |
| rs1198588 | PCR  primers | Forward | TGTAGGAGGCATTTGCAGACAGA |
|  |  | Revered | GGCAAGGGTCGTATGAGATTCAC |
|  | Primer extension | | TAGTAGCCCACAAATCCTCTTCTTTACTTGTTTTTTTTTTTTTTT |
| rs2660304 | PCR  primers | Forward | CTGGTGCTCTCCCAGTGCTTTC |
|  |  | Revered | TCCCAAAGCCGTCTAGGCAAAT |
|  | Primer extension | | AGACTGGGTACCAAGCAGAGAAGTGTTTTTTTT |

## Table S2 Demographic characteristics of sample for analysis of genotype and allele distributions of rs1198588, rs2660304

| Variables | | Patients |  | Controls | Statistical tests |
| --- | --- | --- | --- | --- | --- |
|  |  |  |  |  |  |
| Total Number |  | 1116 |  | 1039 |  |
| Mean Age ± SD (year) | | 34.71 ± 13.64 |  | 34.33 ± 9.44 | t=0.74 *P*=0.46 |
| Gender N (%) | Male | 709(64.53%) |  | 619(59.58%) | χ2=3.56 *P*=0.06 |
|  | Female | 407(36.47%) |  | 420(40.42%) |  |

Abbreviations: N: number; SD: standard deviation.

## Table S3 Genotype of *MIR137* polymorphisms and clinical characteristics of schizophrenic patients

|  | Variables | N | | Genotype | | | | Statistic tests | | *P* | | *P_FDR_* | |
| --- | --- | --- | --- | --- | --- | --- | --- | --- | --- | --- | --- | --- | --- |
| **rs1198588** | |  | | **TT** | | **TA+AA** | |  | |  | |  | |
| Male | |  | |  | |  | |  | |  | |  | |
|  | Age at onset (Mean ± SD, year) | 632/73 | | 34.03 ± 13.00 | | 36.05 ± 13.87 | | t = 1.25 | | 0.21 | |  | |
|  | Family history: n (%) | |  | |  | |  | |  | |  | |  |
|  | + | 91 | | 83(91.21%) | | 8(8.79%) | | χ2 = 2.56 | | 0.11 | |  | |
|  | - | 644 | | 547(84.94%) | | 97(15.06%) | |  | |  | |  | |
|  | PANSS (Mean ± SD, year) | |  | |  | |  | |  | |  | |  |
|  | total score | 589/68 | | 75.35 ± 19.54 | | 74.91 ± 18.95 | | t = 0.18 | | 0.86 | |  | |
|  | P subscore | 596/68 | | 21.51 ± 7.53 | | 20.73 ± 7.57 | | t = 0.81 | | 0.42 | |  | |
|  | N subscore | 596/68 | | 17.10 ± 7.81 | | 17.73 ± 7.64 | | t = 0.63 | | 0.53 | |  | |
|  | G subscore | 596/68 | | 35.43 ± 9.89 | | 35.23 ± 9.51 | | t = 0.16 | | 0.87 | |  | |
|  | BACS (Mean ± SD) | |  | |  | |  | |  | |  | |  |
|  | Digit sequencing task | 250/30 | | 15.82 ± 9.01 | | 17.7 ± 9.18 | | t = 1.08 | | 0.28 | |  | |
|  | Category instances | 255/31 | | 28.78 ± 12.32 | | 28.22 ± 11.89 | | t = 0.24 | | 0.81 | |  | |
|  | COWAT | 246/29 | | 9.46 ± 5.84 | | 9.59 ± 6.15 | | t = 0.11 | | 0.92 | |  | |
|  | List learning | 242/29 | | 21.82 ± 13.48 | | 18.72 ± 14.21 | | t = 1.16 | | 0.25 | |  | |
|  | Token motor task | 250/30 | | 51.10 ± 18.74 | | 42.90 ± 12.79 | | t = 2.33 | | 0.02^*^ | | 0.140 | |
|  | Tower of London | 236/26 | | 7.85 ± 6.38 | | 7.92 ± 5.66 | | t = 0.05 | | 0.96 | |  | |
|  | Symbol coding | 239/25 | | 21.02 ± 12.77 | | 21.36 ± 14.83 | | t = 0.12 | | 0.90 | |  | |
| Female | |  | |  | |  | |  | |  | |  | |
|  | Age at onset (Mean ± SD, year) | 373/33 | | 35.25 ± 14.48 | | 37.87 ± 15.04 | | t = 0.99 | | 0.32 | |  | |
|  | Family history: n (%) | |  | |  | |  | |  | |  | |  |
|  | + | 58 | | 52(89.66%) | | 6(10.34%) | | χ2 = 0.58 | | 0.45 | |  | |
|  | - | 349 | | 300(85.96%) | | 49(14.04%) | |  | |  | |  | |
|  | PANSS (Mean ± SD, year) | |  | |  | |  | |  | |  | |  |
|  | total score | 345/33 | | 81.03 ± 18.19 | | 82.15 ± 17.11 | | t = 0.34 | | 0.74 | |  | |
|  | P subscore | 347/33 | | 21.85 ± 7.26 | | 22.12 ± 7.52 | | t = 0.20 | | 0.84 | |  | |
|  | N subscore | 347/33 | | 19.52 ± 9.63 | | 20.48 ± 9.52 | | t = 0.55 | | 0.59 | |  | |
|  | G subscore | 347/33 | | 38.41 ± 9.41 | | 37.96 ± 7.15 | | t = 0.27 | | 0.79 | |  | |
|  | BACS (Mean ± SD) | |  | |  | |  | |  | |  | |  |
|  | Digit sequencing task | 127/9 | | 12.88 ± 9.31 | | 15.44 ± 10.28 | | t = 0.79 | | 0.43 | |  | |
|  | Category instances | 130/9 | | 26.57 ± 13.06 | | 39.11 ± 14.53 | | t = 2.76 | | 0.01^*^ | | 0.042 | |
|  | COWAT | 127/9 | | 8.94 ± 5.95 | | 9.33 ± 2.69 | | t = 0.20 | | 0.84 | |  | |
|  | List learning | 119/9 | | 23.80 ± 15.10 | | 21.11 ± 13.66 | | t = 0.52 | | 0.60 | |  | |
|  | Token motor task | 131/9 | | 44.03 ± 13.69 | | 48.11 ± 14.01 | | t = 0.86 | | 0.39 | |  | |
|  | Tower of London | 111/9 | | 6.95 ± 6.37 | | 4.56 ± 3.61 | | t = 1.11 | | 0.27 | |  | |
|  | Symbol coding | 109/8 | | 21.48 ± 14.31 | | 21.12 ± 12.55 | | t = 0.07 | | 0.94 | |  | |
|  |  |  | |  | |  | |  | |  | |  | |
| **rs2660304** | |  | | **TT** | | **GT+GG** | |  | |  | |  | |
| Male | |  | |  | |  | |  | |  | |  | |
|  | Age at onset (Mean ± SD, year) | 634/71 | | 34.04 ± 13.00 | | 36.02 ± 13.83 | | t = 1.21 | | 0.23 | |  | |
|  | Family history: n (%) |  | |  | |  | |  | |  | |  | |
|  | + | 91 | | 83(91.21%) | | 8(8.79%) | | χ2 = 2.52 | | 0.11 | |  | |
|  | - | 646 | | 549(84.98%) | | 97(15.02%) | |  | |  | |  | |
|  | PANSS (Mean ± SD, year) |  | |  | |  | |  | |  | |  | |
|  | total score | 591/66 | | 75.38 ± 19.55 | | 74.62 ± 18.82 | | t = 0.30 | | 0.76 | |  | |
|  | P subscore | 598/66 | | 21.48 ± 7.54 | | 20.96 ± 7.49 | | t = 0.53 | | 0.60 | |  | |
|  | N subscore | 598/66 | | 17.15 ± 7.89 | | 17.33 ± 6.83 | | t = 0.18 | | 0.86 | |  | |
|  | G subscore | 598/66 | | 35.45 ± 9.90 | | 35.07 ± 9.43 | | t = 0.30 | | 0.77 | |  | |
|  | BACS (Mean ± SD) |  | |  | |  | |  | |  | |  | |
|  | Digit sequencing task | 251/29 | | 15.75 ± 9.05 | | 18.31 ± 8.71 | | t = 1.44 | | 0.15 | |  | |
|  | Category instances | 256/30 | | 28.67 ± 12.43 | | 29.16 ± 10.85 | | t = 0.21 | | 0.83 | |  | |
|  | COWAT | 247/28 | | 9.44 ± 5.84 | | 9.82 ± 6.13 | | t = 0.33 | | 0.74 | |  | |
|  | List learning | 243/28 | | 21.73 ± 13.52 | | 19.39 ± 14.00 | | t = 0.86 | | 0.39 | |  | |
|  | Token motor task | 251/29 | | 51.09 ± 18.70 | | 42.75 ± 13.00 | | t = 2.33 | | 0.02^*^ | | 0.140 | |
|  | Tower of London | 237/25 | | 7.82 ± 6.39 | | 8.24 ± 5.54 | | t = 0.32 | | 0.75 | |  | |
|  | Symbol coding | 240/24 | | 20.94 ± 12.81 | | 22.25 ± 14.45 | | t = 0.47 | | 0.64 | |  | |
|  |  |  | |  | |  | |  | |  | |  | |
| Female | |  | |  | |  | |  | |  | |  | |
|  | Age at onset (Mean ± SD, year) | 376/30 | | 35.16 ± 14.48 | | 39.33 ± 14.82 | | t = 1.52 | | 0.13 | |  | |
|  | Family history: n (%) |  | |  | |  | |  | |  | |  | |
|  | + | 58 | | 54(93.10%) | | 4(6.90%) | | χ2 = 0.04 | | 0.82 | |  | |
|  | - | 349 | | 322(92.26%) | | 27(7.74%) | |  | |  | |  | |
|  | PANSS (Mean ± SD, year) |  | |  | |  | |  | |  | |  | |
|  | total score | 348/30 | | 80.96 ± 18.14 | | 83.06 ± 17.46 | | t = 0.61 | | 0.54 | |  | |
|  | P subscore | 350/30 | | 21.86 ± 7.25 | | 22 ± 7.62 | | t = 0.10 | | 0.92 | |  | |
|  | N subscore | 350/30 | | 19.50 ± 9.60 | | 20.86 ± 9.90 | | t = 0.75 | | 0.46 | |  | |
|  | G subscore | 350/30 | | 38.37 ± 9.39 | | 38.46 ± 7.29 | | t = 0.05 | | 0.96 | |  | |
|  | BACS (Mean ± SD) |  | |  | |  | |  | |  | |  | |
|  | Digit sequencing task | 129/7 | | 12.93 ± 9.37 | | 15.42 ± 9.43 | | t = 0.69 | | 0.49 | |  | |
|  | Category instances | 132/7 | | 26.79 ± 13.13 | | 38.57 ± 15.91 | | t = 2.29 | | 0.02^*^ | | 0.168 | |
|  | COWAT | 129/7 | | 8.97 ± 5.91 | | 8.86 ± 2.67 | | t = 0.05 | | 0.96 | |  | |
|  | List learning | 121/7 | | 23.91 ± 15.13 | | 18.42 ± 11.45 | | t = 0.94 | | 0.35 | |  | |
|  | Token motor task | 133/7 | | 44.17 ± 13.63 | | 46.71 ± 15.86 | | t = 0.48 | | 0.63 | |  | |
|  | Tower of London | 113/7 | | 6.95 ± 6.35 | | 3.86 ± 2.54 | | t = 1.28 | | 0.20 | |  | |
|  | Symbol coding | 111/6 | | 21.68 ± 14.35 | | 17.33 ± 9.44 | | t = 0.73 | | 0.47 | |  | |

Abbreviations: N: number; SD: standard deviation; PANSS: Positive and Negative Syndrome Scale; P: Positive scale; N: Negative scale; G: General Psychopathology scale; BACS: Brief Assessment of Cognition in Schizophrenia; COWAT: Controlled oral word association test; **P* < 0.05.


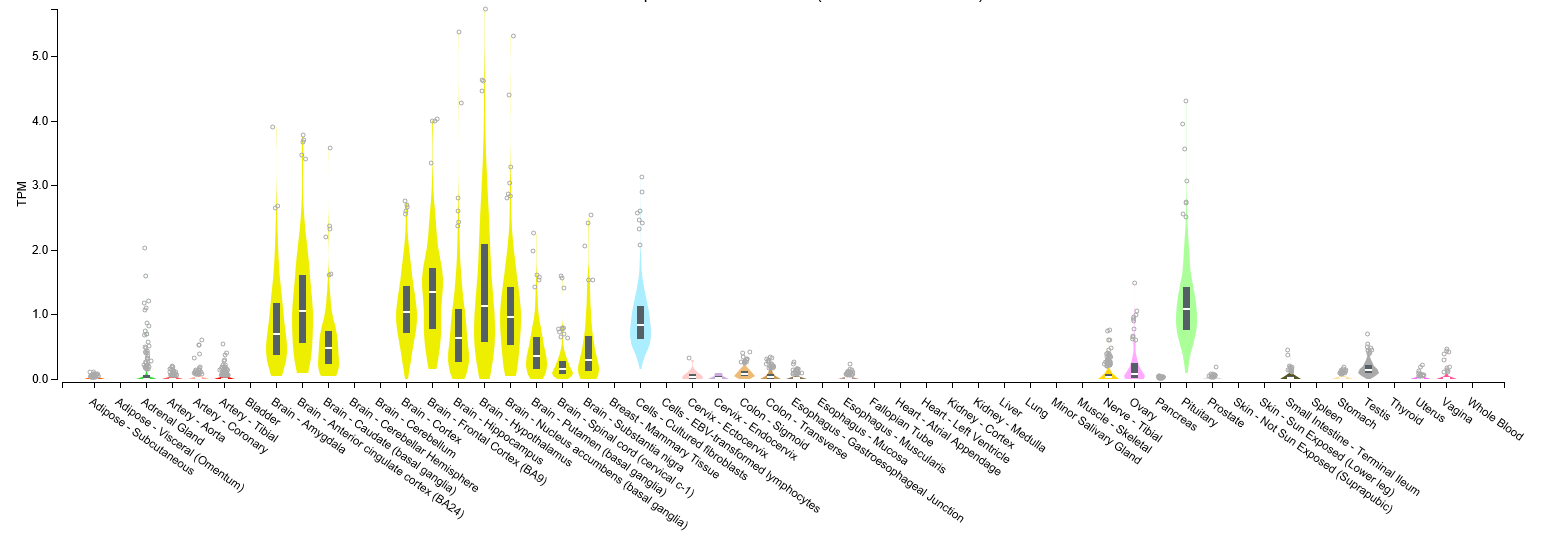


**Figure S1 Gene expression for *MIR137*HG (ENSG00000225206.8)**
